# MAPK3-MYB36-ARF1 module regulates the tanshinone formation in *Salvia miltiorrhiza*

**DOI:** 10.1080/15592324.2024.2391659

**Published:** 2024-08-15

**Authors:** Yongfeng Xie, Hao Liu

**Affiliations:** a College of Environment and Life Sciences, Weinan Normal University, Weinan, China; b College of Agriculture, Ludong University, Yantai, China

**Keywords:** Tanshinone, regulation, MAPK3-MYB36-ARF1, module, *Salvia miltiorrhiza*

## Abstract

*Salvia miltiorrhiza*, known as Danshen, is a traditional Chinese medicinal plant with significant cardiovascular benefits, attributed to its secondary metabolites, particularly tanshinones. Despite their medicinal value, tanshinones occur in low natural abundance, necessitating research to increase their content. This study explores the role of the ARF transcription factor (SmARF1) in tanshinone accumulation in Danshen. Overexpressing *SmARF1* in hairy roots significantly increased tanshinone levels. EMSA and Dual-LUC assays revealed that SmMYB36, a transcription factor interacting with SmMAPK3, binds to and regulates the *SmARF1* promoter. SmMYB36 alone inhibited the expression of *SmARF1* gene, while its interaction with SmMAPK3 enhanced *SmARF1* promoter activity. This MAPK3-MYB36-ARF1 module elucidates a complex regulatory mechanism for tanshinone biosynthesis, offering insights for targeted enhancement of tanshinone content through advanced biotechnological approaches.

*Salvia miltiorrhiza* Bunge, commonly known as Danshen, is a perennial herbaceous plant in the Lamiaceae family. Its dried roots and rhizomes are traditional Chinese medicinal materials, primarily used clinically to treat cardiovascular diseases. The extensive medicinal value of Danshen is mainly attributed to its rich secondary metabolites, and tanshinones are a kind of key active component in Danshen. Tanshinones include cryptotanshinone (CT), dihydrotanshinone I (DT), tanshinone I (TI), and tanshinone IIA (TIIA), which possess various pharmacological properties such as anti-inflammatory, anti-tumor, and anti-myocardial fibrosis effects.^1^ However, the natural abundance of tanshinones is relatively low, making it challenging to meet the growing clinical demand. Therefore, increasing the content of tanshinones has become a crucial direction in Danshen research and development. In recent years, the biosynthetic pathways and regulatory mechanisms of tanshinones have been significantly investigated through molecular biology and biotechnology. The biosynthesis of tanshinones in *S. miltiorrhiza* primarily proceeds through two pathways: the mevalonate (MVA) pathway and the 2-C-methyl-D-erythritol-4-phosphate (MEP) pathway.^2^ Among these pathways, transcription factors play a key role in the biosynthesis of tanshinones,^3^ such as AP2/ERF,^4^ bHLH,^5^ MYB,^6^ bZIP,^7^ and WRKY.^8^

In this study, we generated overexpressing hairy roots of ARF transcription factor in *S. miltiorrhiza*, and identified the roles of *SmARF1* in tanshinone accumulation. The results of HPLC (high performance liquid chromatography) showed the content of CT, DT, TI, TIIA, and TTA (total tanshinones) were significantly increased in SmARF1-overexpressing lines compared to wild-type plants (Figure 1a). Additionally, four MYB binding sites were found in the promoter region of *SmARF1* gene, suggesting tha*t SmARF1* might be regulated by MYB (Figure 1b). To further clarify the regulatory relationship between SmARF1 and MYB, the DNA fragment of the SmARF1 promoter was incubated with the SmMYB36 protein. The EMSA results indicated that SmMYB36 can bind to the *SmARF1* promoter *in vitro* (Figure 1c). In our previous study, *SmMAPK3* is involved in regulating the synthesis of tanshinones in *S. miltiorrhiza*, and SmMYB36 was identified to interact with SmMAPK3 by using yeast two-hybrid (Y2H) assay.^9^ To confirm their interaction, SmMAPK3 and SmMYB36 were fused to the N-terminal and C-terminal of luciferase, respectively, and transiently co-expressed in tobacco leaves. The results indicated that SmMAPK3 interacts with SmMYB36 in tobacco leaves (Figure 1d). Meanwhile, we performed a multiple sequence alignment and constructed a BLAST to compare the similarity between SmMAPK3 and the 17 tobacco MAPKs. The results (Figure S1, Table S1) indicate that there is no identical sequence to SmMAPK3 in tobacco.

**Figure 1.**
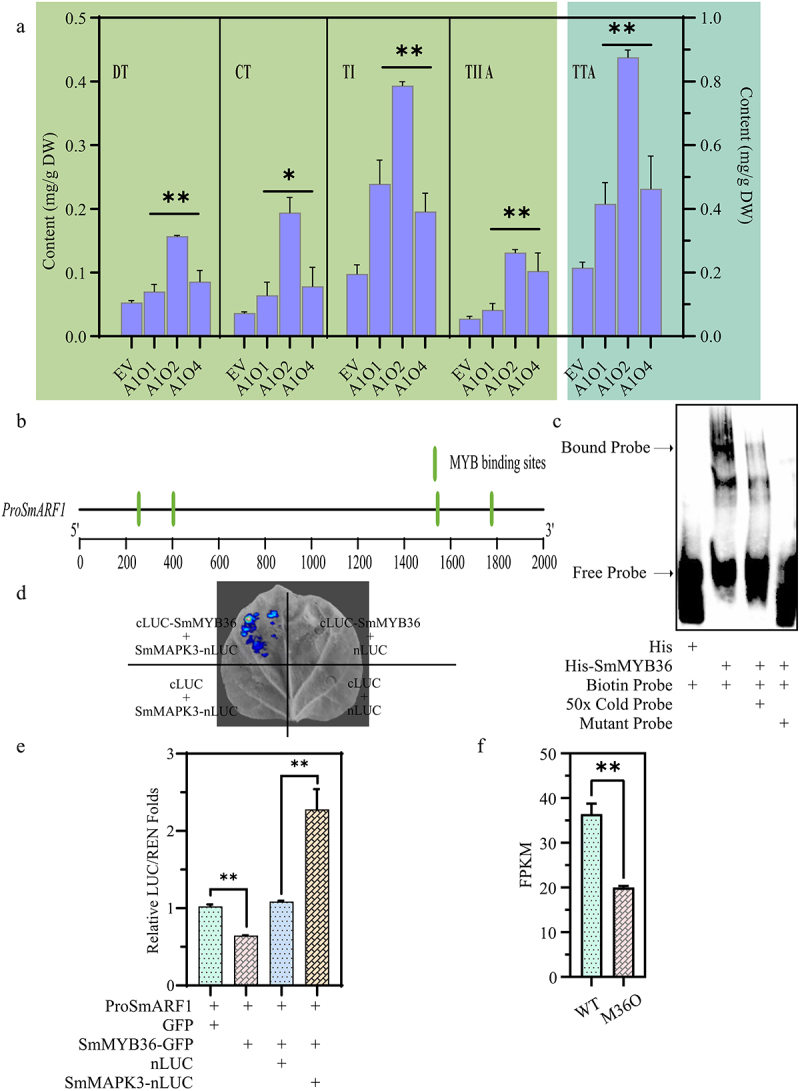
(a) HPLC analysis of the content of cryptotanshinone (CT), dihydrotanshinone I (DT), tanshinone I (TI), tanshinone IIA (TIIA), and total tanshinones (TTA) in wild-type (EV) and *SmMYB36*-overexpressing hairy roots (A1O). (b) Schematic diagram of the promoter for *SmARF1*. (c) EMSA analysis of SmMYB36 and *SmARF1* promoter. (d) Verification of protein interaction between SmMAPK3 and SmMYB36 using LCI assay in tobacco leaves. (e) Dual-luc analysis of SmMAPK3-SmMYB36 and *SmARF1* promoter in tobacco leaves. (f) The expression levels of *SmARF1* gene in the transgenic hairy roots.

To further explore the regulatory mechanism of *SmARF1* gene, a Dual-LUC assay was performed to detect the ability of SmMAPK3 and SmMYB36 to regulate the expression of *SmARF1* in tobacco leaves (Figure 1e). The results revealed that SmMYB36 alone could inhibit the activity of the SmARF1 promoter, whereas the presence of both SmMYB36 and SmMAPK3 enhanced the *SmARF1* promoter activity. Consistently, transcriptome analysis showed that the expression level of *SmARF1* was reduced in *SmMYB36*-overexpressing hairy roots compared to wild-type plants (Figure 1f).^10^ These results suggested that SmMYB36 negatively regulated the expression of *SmARF1*, and the interaction between SmMYB36 and SmMAPK3 could enhance the expression of *SmARF1*.

Interestingly, previous studies have shown that SmMYB36 positively regulates tanshinone accumulation,^11^ and our study also revealed that SmARF1 positively regulates tanshinone accumulation. These were not consistent with our results that SmMYB36 negatively regulated the expression of *SmARF1* (Figure 1e,f). This phenomenon may be due to the absence of SmMAPK3 in tobacco, and SmMAPK3 cannot interactive with SmMYB36 to positively regulate the expression of *SmARF1*. The result that co-expression of both SmMYB36 and SmMAPK3 enhanced the activity of SmARF1 promoter in tobacco leaves also verified this phenomenon. In conclusion, we demonstrated the MAPK3-MYB36-ARF1 module was involved in tanshinone accumulation in *S. miltiorrhiza* and provided valuable insights and references to achieve targeted enhancement of tanshinone content through gene editing and metabolic engineering techniques.

## Supplementary Material

Table S1 .xls

Figure S1.jpg
