# Supplementary figures and images for: MAPK3-MYB36-ARF1 module regulates the tanshinone formation in Salvia miltiorrhiza
Source: Plant Signal Behav. 2024 Aug 15;19(1):2391659. doi: 10.1080/15592324.2024.2391659 (PMC11328878; doi:10.1080/15592324.2024.2391659)

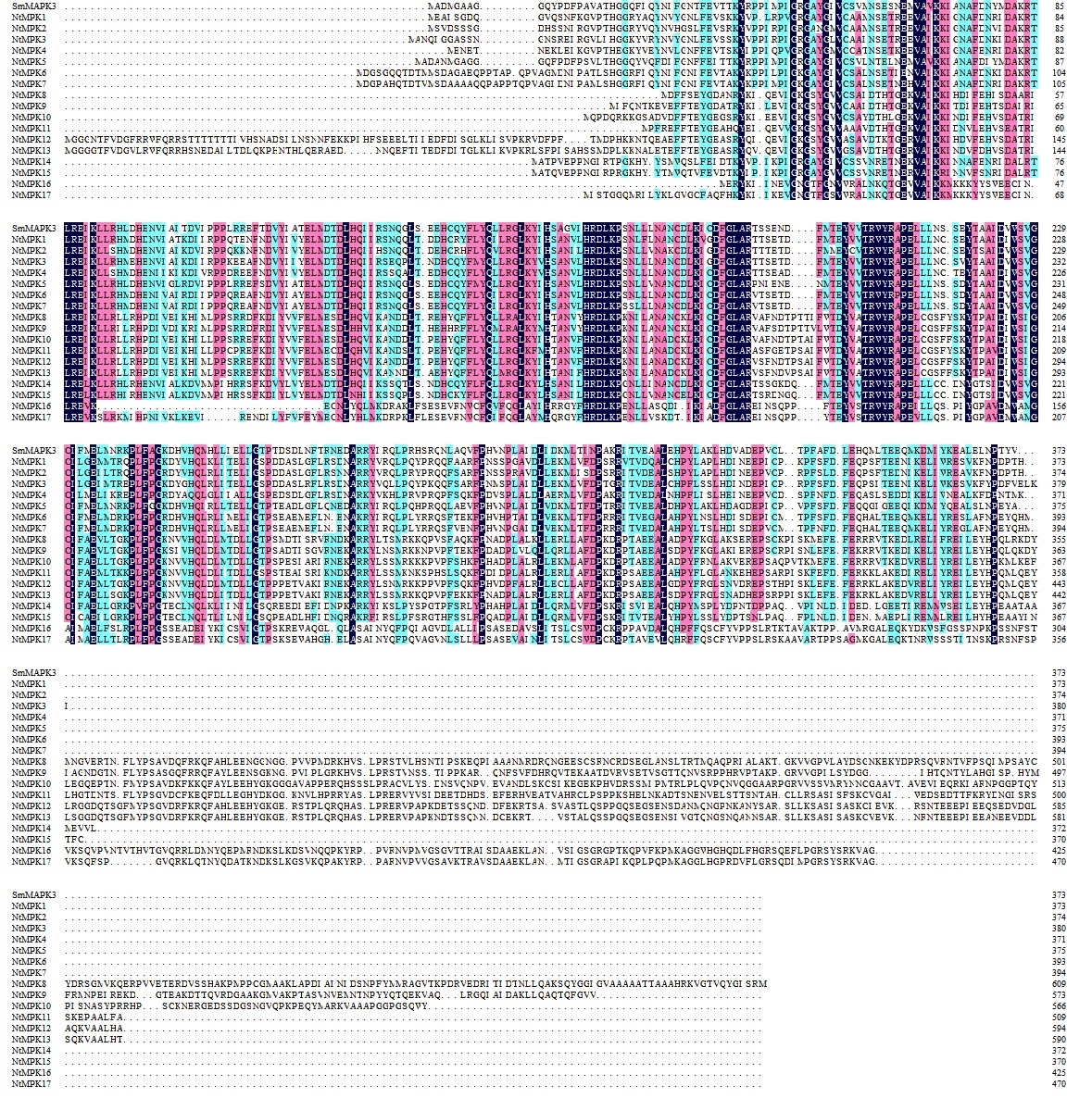

Supplement: Figure S1.jpg [file KPSB_A_2391659_SM0077.jpg]
